# Torture survivors’ experiences of receiving surgical treatment indicating re- traumatization

**DOI:** 10.1371/journal.pone.0287994

**Published:** 2023-10-17

**Authors:** Ana Carla S. P. Schippert, Tone Dahl-Michelsen, Ellen Karine Grov, Bente Sparboe-Nilsen, Juha Silvola, Ann Kristin Bjørnnes

**Affiliations:** 1 Institute of Nursing and Health Promotion, Oslo Metropolitan University, Oslo, Norway; 2 Akershus University Hospital, Oslo, Norway; 3 Institute of Physiotherapy, Oslo Metropolitan University, Oslo, Norway; 4 Faculty of Medicine and Health, Örebro University, Örebro, Sweden; 5 Institute of Clinical medicine, Campus Ahus, University of Oslo, Oslo, Norway; 6 Norwegian University of Science and Technology, Gjøvik, Norway; Walden University, UNITED STATES

## Abstract

Due to the invasive nature of surgical procedures and the involvement of medical personnel, torture survivors may experience re-traumatization during surgical treatment. This study aimed to explore torture survivors’ experiences of re-traumatization during surgical treatment as well as the process by which trauma-related emotions and responses are evoked during surgical treatment for torture survivors. Eight men, aged 45 to 72, from four different countries, who have lived in Norway for 6–40 years, were recruited. We assessed torture and surgical care experiences through in-depth interviews, and the data were analyzed using thematic analysis, resulting in five themes: (1) Interactions with healthcare providers, (2) Reactions during treatment, (3) Triggers causing re-experiences, (4) Avoidance, and (5) Suggestions to healthcare providers. In this study, survivors reported challenges receiving surgical treatment, indicating re-traumatization and difficulty returning to daily life following treatment. Participants reported little collaboration in care-related decision-making processes, lack of recognition of torture by healthcare providers involved in surgical care and experiencing healthcare professionals’ attitudes as a source of perplexity, frustration, and despair. Exacerbation of torture memories throughout treatment and re-experiencing of trauma symptoms aggravated these difficulties. Our findings suggest that surgical treatment can remind torture survivors of the traumatic aspects of torture, eliciting strong reactions and feelings like those experienced during torture.

## Introduction

Survivors of torture are susceptible to re-traumatization during treatment, and the more invasive the medical procedures, the greater risk of re-traumatization [1–3]. Re-traumatization during psychological treatment has received more attention in scientific research than re-traumatization during treatment in somatic healthcare departments, such as surgical departments [2, 4, 5]. Because surgical methods are invasive and performed by medical personnel, surgical treatment may contain elements that remind survivors of torture methods causing re-traumatization [2]. Hospitalization, relation with healthcare providers, invasion of the body by medical examination or treatment, and loss of privacy and control due general anesthesia can all trigger feelings or sensations associated with previous torture trauma [1, 2, 5–7]. Surgical procedures can also cause neurological changes, heightening the intensity of the reactivation of past torture trauma, manifested as emotional and behavioral responses [2, 8]. General anesthesia has been linked to feelings of loss of control, similar to feelings experienced during torture [9, 10], and the use of local anesthetics can cause sensations of not feeling a part of the body, which have been linked to damage after hanging during torture [5]. Following general anesthesia, torture survivors may experience dissociated flashbacks, altering their mental status in the post anesthesia care unit [10]. Fear of reliving torture trauma symptoms during surgical care can cause survivors to avoid treatment [2, 5], resulting in health deterioration. This fear is also related to the fact that torture is a person-to-person violent act, which destroys the survivor’s ability to have trusting relationships with other people in general [7], and in some cases, they do not trust health personnel because of their involvement in torture [11, 12]. As trauma is defined as the direct experience, witnessing, exposure to actual or threatened death, serious violence/injury, disaster, or sexual violence [13] and traumatic exposure can occur via direct, indirect, or learned experience, torture trauma can result from both being tortured and witnessing the torture of others [14]. Traumatization occurs when something "becomes too much" and invades one’s personal boundaries, causing overwhelming anxiety that cannot be described. The individual is overwhelmed by external and internal impulses and cannot comprehend or make sense of what is happening [7]. There is substantial evidence of a dose–response relationship between cumulative war trauma and torture and the development and maintenance of post-traumatic stress disease (PTSD) [15]. Refugee torture survivors are approximately four times more likely to suffer from PTSD and approximately two-and-a-half times more likely to suffer from depression than other refugees [16]. However, not all refugees with a history of trauma exhibit mental health symptoms [17] demonstrating posttraumatic resilience as the capacity to recover from and positively adapt to traumatic experiences [17].

As an extreme stress experience torture harms the stress response system and has long-term neurobiological, physiological, and psychological consequences. These neurobiological stress responses are described as part of a universal biological response shared by individuals, cultures, and mammalian species [18]. Because re-traumatization is a reactivation of trauma, neurobiological, physiological, and psychological signs or symptoms may appear as follows: Feelings of losing control combined with medical procedures can cause emotional harm and dysregulation in torture survivors, while “psychological preparedness” to the procedures can act as a protective factor against dysregulation [2, 19]. Survivors describe re-traumatization as feeling skinless and exposed [20]. Any sensation reminiscent of torture can cause “flooding,” which is defined as strong sensations that appear too quickly and overwhelm the body, indicating that the patient has difficulty moving in and out of the situation or interaction [20]. Because of fear, the central nervous system can become dysregulated during re-traumatization [21, 22], and the body may struggle to return to a pre-trauma state of balance and stability. As a result, the survivor’s subjective sense of safety and well-being may be jeopardized. Individual responses to dysregulation can include dissociation, hyperarousal, aggression, avoidance, or dependence [21, 22].

What can cause re-traumatization is related to the patient’s experiences [23], relations [7] and situations resembling torture [2, 5]. As a result, healthcare professionals need to know what torture is, the most common methods of torture, and how torture impairs the survivor’s ability to form trusting relationships with others [7].

The United Nations Convention Against Torture and Other Cruel, Inhuman or Degrading Treatment or Punishment (UNCAT) defines torture as “an intentional act of physical or psychological harm inflicted on a person for the purposes of obtaining information, punishment, intimidation or discrimination of any kind by a person in or acting for a person in an official capacity” [24]. According to this definition, torture occurs when the following criteria are fulfilled: (i) strong pain or suffering is inflicted, either physically or mentally; (ii) forced confessions, information, or punishments are sought; and (iii) a public authority carries out, encourages, or consents to the use of torture [24]. The practice of torture is unethical and prohibited by international and national laws [25, 26], but it persists, and torture survivors are usually exposed to repeated and varied acts of violence or witnessing its infliction on others, causing fright due to threat to life, health, and sanity. Acts of torture spread distrust among survivors and between them and their friends, family members, and the community [7, 27], destroying important and vital relationships for recovery. The primary objective of torturers is to gain power over others, as well as to silence and intimidate them. Torture has been utilized to control individuals, communities, and even entire nations. Numerous individuals were rendered speechless as a result of the fear that it generated [7, 28, 29].

Symptoms seen in torture survivors’ bodies include difficulty performing simple movements such as walking and running, high levels of body tension, increased muscular tension affecting physical alignment and standing ability, and constricted breathing [20]. Concussions, pain, scars, deformities from healed fractures, nerve and vessel injury to tendons and ligaments, organ dysfunction, mutilation, dislocations, cuts, stabbing and gunshot wounds, and many other health impacts can result from torture [30–32]. Many of these sequels force survivors to seek medical attention for the rest of their lives. Surgical treatments, for example, are frequently required [2, 33], and torture survivors are both treated and researched in healthcare institutions [34, 35].

According to studies on the treatment of sequels after torture, patients who have survived torture frequently report that healthcare services do not meet their health needs [34, 36–39]. Other issues associated with torture trauma include the occurrence of flashbacks triggered by stimuli reminding survivors of the original trauma and manifesting as if they are occurring in the present. Evidence shows that triggers, such as sounds like crying, screams, slamming doors, explosions, smells associated with burning, visual triggers such as wall color, uniforms, blood, and other triggers like small rooms, darkness, or strong light can lead to avoidance of situations in the medical context containing these triggers. In addition, they can induce hypervigilance, fear, and nightmares [5, 40]. However, patients are rarely asked about their torture experiences or how they dealt with these triggers during treatment [34, 41].

As a result of human-induced trauma, torture leaves a legacy of attachment conflicts and difficulties [42], and survivors may have difficulty establishing a trustful relationship with healthcare providers [7, 43]. The reflective response of healthcare providers to the patient-reported torture trauma is critical in creating a therapeutic climate [7, 42]. The trauma response is the reflexive response of the body to a perceived threat. This "threat" need not be of a physical nature. It can also be a source of stress, such as the surrounding environment or interactions with healthcare providers [44, 45].

The mind and body of humans are remarkably resilient because they evolved with a preprogrammed desire to avoid danger though the body’s natural defense mechanism (fight, flight, freeze, fawn, flag, and faint) against mental or physical threats. The fight response occurs during the alarm phase, when adrenaline levels are highest. Fight types believe that power and control can provide safety as a trauma response, and they may respond to stressors with unacceptable anger and aggression. Torture survivors may awaken from general anesthesia with an extremely hostile disposition as a fight response. They may also exhibit rapid breathing, rapid heartbeat, perspiration, alertness, and anxiety. The patient may experience emotional outbursts or misbehavior [45]. A freeze reaction is a camouflage response in which a survivor hides, isolates, and dissociates to avoid additional stress. The patient does not respond to healthcare professionals’ communication, avoids eye contact with healthcare professionals, and remains silent. Additional indicators of the freeze response can occur with heart and respiratory rates dropping dramatically. The flight response is an acute stress response that occurs immediately after the "freeze" state. Adrenaline-seeking behaviors, overworking, and obsessive and compulsive behaviors are used to escape from the trauma or the trigger. The patient avoids potentially trigging situations as well as interactions with healthcare providers.

As important as knowing how to prevent re-traumatization is the ability to recognize the signs and symptoms of retraumatization, as patients may require additional care to be able to manage the feelings and reactions associated with re-traumatization [10].

Healthcare providers working in somatic departments and particularly surgical departments are extensively trained in the technical care of the wounded body, but they are not necessarily prepared to diagnose, prevent, or treat the range of mental health issues that frequently follow injuries caused by trauma [46].

Re-traumatization of torture survivors during surgical treatment has been discussed in the literature [47, 48]. However, systematic knowledge synthesis and research focusing on survivors’ self-reported experiences are lacking. According to a systematic review summarizing qualitative research evidence on torture survivors’ somatic healthcare experiences, torture survivors do not receive adequate healthcare and may face challenges during treatment, which can lead to re-traumatization. Although the review search resulted in more than 3,000 articles, only eight were included [49]. Therefore, new knowledge based on research findings is required to improve health services for torture survivors and to prevent re-traumatization during treatment for somatic diseases.

## Aim of the study

This study is a part (stage 3) of a project [50] aimed to development and evaluation of guidelines for the prevention of re-traumatization of torture survivors during surgical care. The aim of the present study was to gain knowledge about torture survivors’ experiences of receiving surgical treatment in various departments, such as operating rooms, intensive care units, and outpatient clinics as well as to identify potential triggers evoking trauma-related emotions and responses involved in the re-traumatization process. We also present aspects suggested by the participants to improve the quality of care and prevent re-traumatization during treatment.

## Methods

Individual qualitative in-depth interviews are the preferred research method in order to gain an in-depth understanding of participants’ experiences, especially when these experiences are considered to cause someone to feel sensitive and vulnerable [51]. Accordingly, individual in-depth interviews were the obvious choice in this study, which included interviews with eight torture survivors who had received surgical treatment in various Norwegian healthcare contexts.

This study’s recruitment strategy is described in Stage 3 of the study protocol [50]. Asylum institutions, refugee health centers, district psychiatric centers, and university hospital surgical departments in Norway recruited participants. We also contacted the Health Centre for Undocumented Migrants, Red Cross Office, Church Foundation (Stiftelsen Kirkens Bymisjon), and Migrations Health Centre for recruitment.

The study was approved by the Norwegian Committee for Medical and Health Research Ethics. By informing potential recruiters about the project, we emphasized that participants should not only comprehend the presented information, but also be able to recognize the personal effects of research participation on the participant’s life. Two participants were recruited by their therapists, and two by their physiotherapists, based on their assessment of consenting capacity. Two participants were recruited through organizations with the assistance of healthcare providers who assessed the consenting capacity of each participant prior to the interview. The final two patients were recruited through private network, and relatives assessed their consenting capacity.

To explore survivors’ experiences of receiving surgical treatment, we prepared an interview guide based on findings from a literature review [49]. The interview guide included eight thematic areas for exploration of torture and treatment experiences: (1) torture story and torture, (2) hypersensitivity after torture (distrust, vulnerability, powerlessness, loss of control, low self-esteem, and difficulty in self-advocating, (3) surgical treatment, experiences, and feelings, (4) hypersensitivity for threats to safety related to surgical treatment, (5) triggers and re-traumatization, (6) reactions indicating re-traumatization, (7) avoidant coping, and (8) expectations and advice to healthcare providers.

The interview guide, presented as a S1 File, was semi-structured, implying that the topic was covered, but the interview was more conversational in nature, and the guide was not necessarily followed point-by-point.

Further, in line with recommendations by Ojeda and colleagues (2011), the interview began with demographic questions to help the participants feel at ease and confident about their ability to answer questions [52]. This was followed by a question inviting participants to discuss their torture experiences if they wished and in a manner that was comfortable for them. Additionally, they were told to take as much time as necessary [53].

The first author conducted seven interviews between May 2021 and August 2022 and the eighth interview in January 2023. The interviews were conducted in various parts of Norway and in settings chosen by the interviewees. An interpreter was offered to all participants, but only three requested one. Two participants wanted the presence of their therapist during the interview, and two desired the presence of their wives. All interviews were audiotaped with the participants’ permission, and the transcripts were stored in accordance with the regulations of the Norwegian Centre for Research Data and the research and storage guidelines of Oslo Metropolitan University. The interviews lasted between one and two and a half hours. The interviews were conducted in one sitting with only necessary breaks. Some participants accepted the offer of breaks, while others wished to continue without interruptions.

The participants were also asked to complete a questionnaire (S2 File) based on the Harvard Trauma Questionnaire (HTQ), a cross-cultural screening instrument developed 25 years ago to document trauma exposure, head trauma, and trauma-related symptoms in refugees [54]. The participants could complete the questionnaire after the interview or later. The responses to this questionnaire were used to supplement the data about torture, particularly for participants who did not discuss their torture experiences during the interviews.

### Ethical considerations

This study followed the Helsinki Declaration [55]. In May 2021 (#227624), the Regional (South-East C) Committee for Medical and Health Research Ethics, the Data Protection Official for Research, and Akershus University Hospitals’ data protection officer (PVO) approved the project. We informed the participants, relatives, and interpreters of the confidentiality of the interviews’ and emphasized that participation was entirely voluntary. The written consent form (S3 and S4 Files) was read aloud, and both, participants and relatives were encouraged to ask additional questions about the interviewing procedure. Just prior to the interview, the participants and the relatives signed a consent form and were informed that fictitious names would be used to protect their anonymity when the data were presented. To prevent backward identification, events were described so that individuals were de-identified and the significance of the event was highlighted. Before the interviews, the participants were informed about the aim of the study to minimize disappointment and mistrust [56, 57]. The vulnerability of this group of participants was taken into account as well as who they could contact if they experienced strong emotions and reactions after the interviews [35, 58]. The S5 File describes the protocol developed by the research team to assist participants in dealing with strong reactions if they occurred during or after the interviews. In addition, stress, trauma, and grief specialists (AKB, EKG, and BN) were available for any necessary follow-ups.

To ensure that no strong reactions remained, the interviewer called each participant the day after, one week after, and one month after the interview. One participant called the interviewer twice to add forgotten information.

### Participants

We presented the data using fictitious names. The participants were all tortured and came from different countries in South America and the Middle East, and they have lived in Norway for 7–40 years. They were all treated in at least one surgical department in Norway. The overview of the participants, the described torture methods, context and sequels, type of treatment, treatments context, and healthcare providers involved in the treatment are described in S1 Table.

### Analysis

Using thematic analysis (TA), the data collected from the survivors were explored to uncover essential themes. TA methodically finds, organizes, and analyzes dataset themes, helping researchers understand common ideas and experiences by concentrating on meaning across datasets. In addition, TA does not identify individual meanings and experiences within a single data item. Thus, this strategy identifies and interprets commonalities in how a topic is discussed or written about [59]. The six steps of inductive thematic analysis: (1) familiarizing yourself with your data, (2) generating initial codes, (3) searching for themes, (4) reviewing themes, (5) defining and naming themes, and (6) producing the report, as recommended by Braun and Clarke, were used in the analysis to help draw conclusions from the data [60, 61].

To gain familiarity, the interview transcripts were read several times before a list of codes was created from the data. ACS used line-by-line coding to retrieve codes and categorize transcripts into themes and subthemes while regularly consulting with AKB, TDM, and BN and revising. In order to create an initial thematic map with all pertinent themes and supporting codes, themes, and subthemes were compared between the participant datasets and the complete dataset. The themes were evaluated and improved before they were finally defined and named. This required creating a final thematic map to create a comprehensive overview of the prevailing themes. To demonstrate the quality of the thematic analysis, we followed the criteria recommended by Braun and Clark [62]. The transcriptions were verified against the audio recordings. Each data item received equal consideration during the coding process, and there was a balance between the data (i.e., quotations) and analytic narrative. The majority of respondents were represented by a single quotation, no respondent was represented by more than three quotations, and the researcher was active in the research process [61, 62].

## Results

The participants were encouraged to discuss torture in any manner and in any terminology they wished. Six participants recounted their stories in detail, whereas two disclosed only a few specifics regarding ill treatment in their home countries.

**Fig 1:**
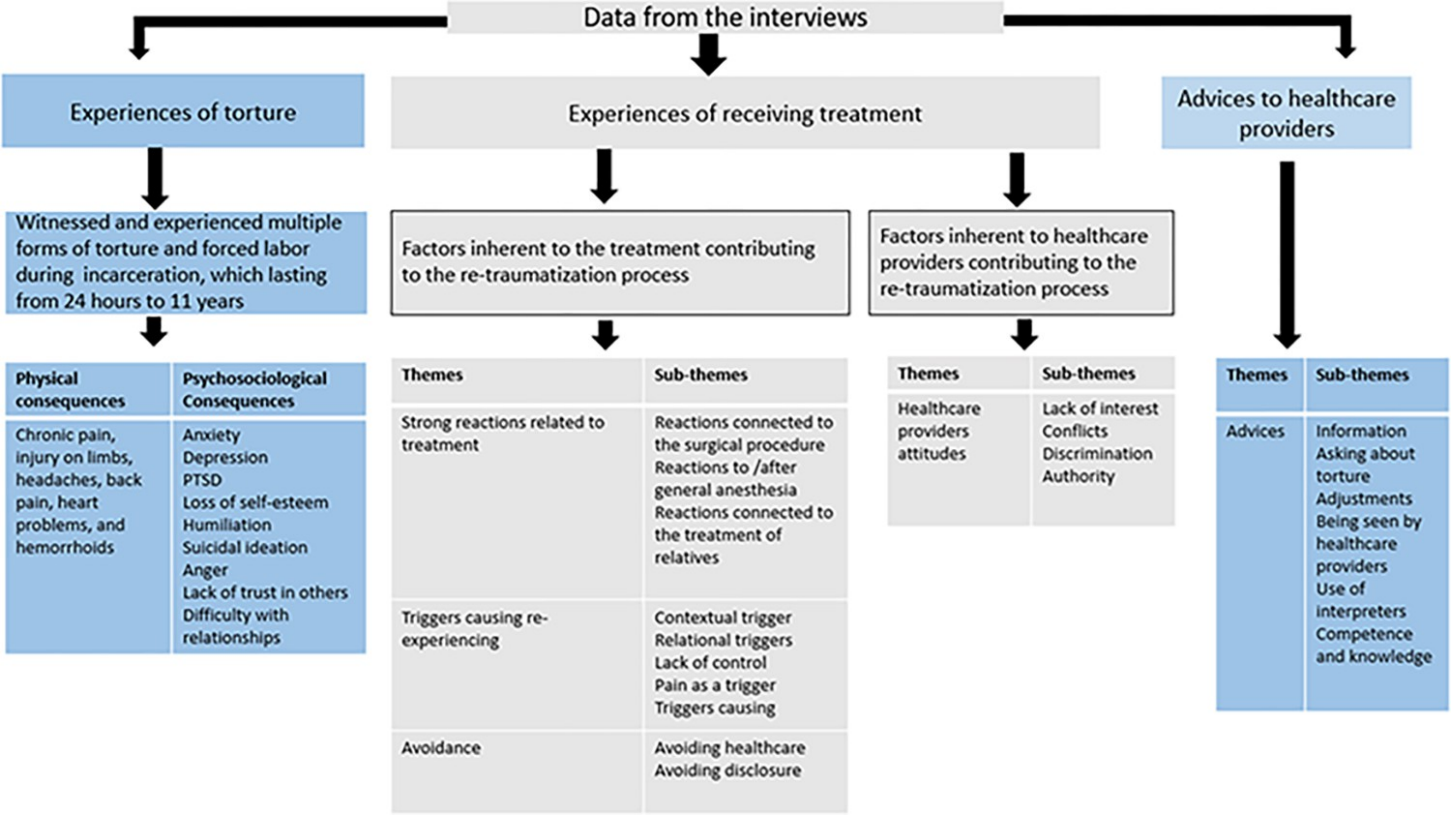
Presentation of the data.

As previously described, the re-traumatization process is complex [1], and in order to comprehend it, it is necessary to try to understand the whole picture as completely as possible of what causes re-traumatization in the context, and in the patients’ relationships with healthcare providers [1]. Leaving out one of these components prevents comprehension of the entire process. Consequently, the data are presented in its entirety, as depicted in Fig 1.

### Description of the torture experiences and their effects

The participants reported that they had been targeted by perpetrators for a variety of reasons, including their ethnicity, religion, occupation, involvement in the military, and political activities, either on their own or on the part of their family members. They were subjected to multiple forms of torture and forced labor during their incarceration, which lasted anywhere from 24 hours to 11 years. During this time, they both witnessed and experienced various forms of torture. Chronic pain, injury on limbs, headaches, back pain, heart problems, and hemorrhoids were some of the many adverse physical effects of their ordeal, which included torture. Participants reported a wide range of negative psychological effects, such as symptoms of anxiety, depression, and (PTSD), as well as loss of self-esteem, humiliation, suicidal ideation, and anger. The emotional disconnection from loved ones, isolation, decline in social status, and unemployment were some of the social effects. Most participants cited the difficulty in establishing trustworthy relationships with others, including healthcare providers, as one of their primary concerns.

### The experiences of receiving surgical treatment

The participants were treated in surgical departments at various healthcare institutions throughout Norway. Five of them received surgical procedures, while the other two were evaluated for surgery in a surgical outpatient clinic but chose not to go through with the procedure. Three of the participants underwent heart surgery. Every surgical procedure required the participation of surgeons, anesthesiologists, and nurses.

All participants expressed deep appreciation for the warm welcome they received in Norway, where they have lived in peace. Despite certain difficulties in adapting to a new culture, participants demonstrate a high level of resilience as they continue with their lives, education, employment, and community support. Participants emphasize their belief that the Norwegian health care system is an effective system that provides treatment options. During the interviews, the participants had difficulty criticizing health professionals, and whenever they described difficult situations, they also attempted to explain where they were attempting to excuse health professionals and blame the system for their failures.

One participant, speaking of his experiences receiving hemorrhoid treatment without painkillers and enduring pain, attempted to absolve the professionals involved by stating that they could not have anticipated that he would be unable to withstand the pain. In general, participants emphasized the importance of sharing their experiences due to the possibility of influencing a change that can result in better care for survivors of torture. According to their assessment, Norway’s healthcare services for torture survivors have significant room for improvement.

### Themes and subthemes

Five major interconnected themes emerged from the experiences of torture survivors receiving treatment and being examined in surgical departments: interactions with healthcare providers, reactions to the treatment, triggers causing re-experiencing, avoidance, and advice to healthcare providers.

#### 1. Interaction with healthcare providers

In this theme, the focus is on how the participants experienced the interactions with healthcare providers during treatment. This includes descriptions of healthcare providers’ behaviors and their impact on patients’ reactions and treatment.

*a) Participants’ experiences of their healthcare providers’ behaviors*. The participants expressed that healthcare providers somehow did not care about their torture or premedical conditions and that they had to fight for their healthcare rights. One of the participants said that even after he had been hospitalized 12 times for surgical procedures, no one questioned him about the torture. He described that his experience of torture was disregarded by hospital personnel. In addition, several participants said that healthcare professionals ignored their pain. Despite being the only patient in the surgical emergency department, one participant said that he had to wait several hours, although he had severe pain. He interpreted this as a lack of appropriate care and explained that all he needed was medical care that met his needs, not additional attention.

Notably, the participants’ experiences of the interactions with their healthcare providers reminded them of their torturers who claimed to get information from the victims. Several participants characterized what they experienced the healthcare providers’ attitudes as a source of friction during the encounter, and this made them in an unimportant position. In the end, this experience could also result in a decline in surgery. Participants expressed that the combination of being unimportant and their perception of healthcare providers’ difficulty to comprehend their situation of living alone without relatives to provide support following surgery led to a decline in surgery (expression from two participants).

*Jamil*: “*A doctor*, *[surgeon] how can I say*, *for example*, *when you have a conflict with a person and you do not want to talk to that person*, *it was like that*. *I thought such a person is going to operate on me*. *What should I do*? *No*. *No*!*”*

Participants expected healthcare providers to inquire about and be interested in their medical history; instead, they were met with hostile questions and accusations of systemic exploitation:

*Nasab*: “*They thought that I was pleased to be in the hospital so as to sleep in the hospital*, *but I informed them that I had never been admitted to a hospital before*. *They had accused me of simply being in the hospital to sleep and consume the food*.*”*

In the interviews, all participants expressed disturbing feelings toward people who represented their home countries’ political systems, and they saw healthcare professionals who they now met in the healthcare system in Norway as part of the system, especially when they reacted in an authoritarian manner.

Furthermore, the participants described instances in which they felt discriminated against by healthcare professionals due to their non-Norwegian nationalities. The participants compared the lack of interest in and discriminatory and authoritarian attitudes of healthcare providers to those of torturers and healthcare workers who collaborated with torturers.

*Tiam*: “*I had a mental vision of the doctor in prison treating me like a zero*. *Now he’s here*!*”*

In addition, a comparison was made between healthcare professionals who did not offer them treatment and physicians who assisted torturers. Participants reported that recurrent negative experiences, particularly with the attitudes of healthcare personnel, reinforced their feelings of anxiety and despondency during and after treatment. These severe reactions may last for days, causing significant discomfort and day-to-day difficulties. Strong experiences of fear and insecurity were also associated with the inability of healthcare professionals to communicate. What the participants perceived as inadequate communication skills were frequently associated with a lack of information concerning procedures, routines, and medications, resulting in unpreparedness, despondency, and anger.

*Bashir*: “*It was the one time I felt that I was not treated as a human being*, *not even as an animal*, *because I was sleeping in my room and at one o’clock in the morning*, *a little after one*, *they took me out of the room to another room without reason*. *And then the anesthetist and nurse*, *without saying anything*, *started to cut my neck and put a tube—a catheter—in my neck*.*”*

Another participant described intense feelings of uneasiness owing to information that he believed to be incorrect from healthcare providers, and he felt more like a case than a patient after being checked by many different doctors.

*Nasab*: “*I felt like an experiment in which people just try things on me*.*”*

Participants recalled caregiver relationships during treatment, and emotions like those experienced in prison and under torture as coexisting with treatment memories. Nonetheless, memories and emotions associated with the secrecy surrounding the history of torture predominated. When healthcare professionals did not inquire about their past before or during treatment, participants reported neglected. Healthcare professionals’ behaviors were cited as a factor eliciting the same sentiments as torture because their torturers were indifferent to their personhood.

*b) Lack of trust in healthcare providers*. Although the vast majority of participants believed that healthcare providers genuinely wanted to help them, some reported feeling anxious in their presence. Others claimed that because healthcare professionals were among those who inflicted pain in prison, they reminded the patients of their torture experiences. Because of the brutality of torture, all participants reported having difficulty trusting others. One person’s faith in others was shattered in prison, they noted. The participants had little trust in doctors from their home country, and one participant did not dare to seek medical treatment for his torture wounds in his home country due to the affiliation of the healthcare system with the dictatorship. Another participant stated that his experience as a healthcare professional gave him confidence in other healthcare providers. When asked if he trusted everyone who treated him, he said, “Yes! Because I worked in healthcare” "(Antonio).

Participants who lacked trust in healthcare professionals attributed it on their behavior and attitudes, and one participant associated it on the insensitivity of healthcare providers and the system. He criticized healthcare personnel’s egoism and lack of altruism and said the following about healthcare practitioners:

*Jamil*: “*When they finish their degree*, *they make promises to the university*. *However*, *they are concerned only with their position and reputation*. *Provide us with humanitarian aid*, *just like Doctors Without Borders*!*”*

Participants recalled treatment-related incidents reflecting lack of trust in medical experts due to inconsistent information provided by healthcare providers.

*Nasab*: “*It is normal for me to react by leaving when a doctor tells me one thing*, *another doctor tells me something else*, *and so on*, *and so forth*.*”*

Furthermore, he stated that he did not fully trust the students (who he met in the healthcare system) and would rather avoid them. This lack of trust made him feel unsafe and angry, and reminded him of his previous torture experiences. Participants described how painful medical procedures without anesthetics affected their perception of healthcare providers, causing them to doubt their concern. They believed this would lead to a loss of confidence in healthcare providers.

*c) Healthcare providers’ lack of knowledge*. Participants expressed dissatisfaction with the treatment because they felt their concerns about torture had not been responded to. It was revealed that healthcare personnel’s responses to patients’ health issues, such as pain, were experienced as limited or inadequate. The participants confirmed that healthcare practitioners’ attempts to explain symptoms were not as expected due to their lack of awareness and knowledge about the consequences of torture. They stated that lack of knowledge made them feel uneasy throughout treatment, and they compared these experiences to those they felt while incarcerated. Participants stated that having more knowledge about torture would allow healthcare professionals to provide more appropriate care and make patients feel safe:

*Tobarak*: “*It would have been preferable because they could have paid more attention and treated the patient in a calmer and softer manner with soft hands*. *It would have been nice to create the impression that torture patients are not like everyone else*. *You must exercise caution when conducting your investigation*. *Softer as well*. *So*, *it would have definitely been advantageous to have the knowledge*.*”*

Another participant described his struggle with severe back pain following an operation to repair back injuries caused by torture. The back pain increased after the surgery, putting him in the same condition as if he had been tortured with torture-related thoughts and feelings of fear and anxiety.

*Ramin*: “*The doctors were unaware*, *but the operation was akin to being tortured due to the pain that intensified after the procedure and lasted for 18 months*.*”*

After a few years, the screws in the back were removed due to pain. After that, the pain nearly vanished, life dramatically improved, and all the torture-related thoughts and memories presenting daily challenges became less intrusive. Ramin believed that what he was subjected to (the surgery itself, the pain, and the intrusive torture-related memories) was due to the healthcare providers’ general lack of knowledge regarding the effects of torture and how to treat injuries sustained during torture.

#### 2. Reactions during treatment

This theme addresses participants’ reports of their experiences during and after treatments or examinations in surgical departments as well as their reactions following general anesthesia.

*a) Reactions connected to the surgical procedure*. Several participants mentioned facing obstacles that evoked powerful emotions during treatment under local anesthesia. It was explicitly stated that the use of sharp instruments and needles were some of the causes of their reactions. When asked about his experience with a needle, Tiam said, “Wow! Frankly, I was taken aback by it.” He recounts that both he and the surgeon were surprised by his intense reaction. Due to this response, the symptoms associated with torture resurfaced while receiving care. He felt ashamed and disappointed in himself as a result. Tiam claimed that these striking responses to the local anesthetic were quite strong during his first surgery, but not during the second procedure.

Several participants reported feeling sick, sweating profusely, and having difficulty breathing before and after treatment. One participant reported feeling out of control, like an “alcoholic,” and not liking himself after local anesthesia surgery. He reported postoperative confusion and disturbed sleep, and he felt like his personality had changed completely. He went to see a psychologist for help because his anxiety was so strong.

*Jamil*: “*I needed a psychologist because I wasn’t myself*. *I lost the balance with sleep*, *couldn’t eat*, *my mouth was dry*. *For a long time*!*”*

Another participant claimed that surgery caused post-traumatic stress disorder (PTSD) because it reactivated experiences from the past. His symptoms manifested in both “the head and the body,” he claimed. In addition, his body failed him while he was receiving treatment. He described how the entire situation triggered a series of uncontrollable bodily reactions. He did not realize what it was at first, but he soon realized it. He expected and was prepared for PTSD because several of his comrades had developed it because of torture, so he had waited for his turn. It appeared after his heart surgery. As a result, he became sensitive to healthcare issues and felt disconnected from himself. Following surgery, he experienced strong emotions and the involuntary appearance of memories of past traumatic events.

Ramin had back surgery on several occasions. He described in detail how he reacted when he was placed on the operating table, and a special device was used to keep him in the correct position during surgery. He explained that it was the same position he was forced to take during torture. He entered a mental state that made him feel like he was in prison. He was expecting to be punched under his feet. He said he could almost feel the blows of torture.

*b) Reactions to and after general anesthesia*. Memory loss during hospitalization and recovery at home was reported by all participants who underwent general anesthesia. The agony caused by anesthesia was excruciating when they awoke, and they described experienced as if they were approaching new restrictions and feeling different, more sensitive and defeated. There were violent reactions in the postoperative department as well as difficulty coping with panic attacks at home the night after surgery. One participant claimed that the medical staff informed him that when he had woken up from anesthesia, he was restless and attempted to remove wires and tubes. He had no recollection of anything. Participants expressed anger, particularly during the first night after surgery, explaining that memories of torture exacerbated feelings of anger after surgery. After receiving general anesthesia, participants described transformations similar to those experienced following a nightmare about torture or the torture self. As a result, the experiences were tainted with negativity, leading to a lack of comprehension of what had occurred. Participants expressed difficulty adjusting when confronted with the perception that something had changed.

#### 3. Triggers causing re-experiencing

This section examines the participants’ impressions during treatment they received, such as visual, aural, or bodily sensations as well as the relational aspects of the clinical encounter that generated memories, emotions, and responses related to torture in participants. It is plausible that there is a connection between the types of torture to which the participants were subjected and the conditions that can trigger a reoccurrence of the traumatic consequences of torture when the patient is undergoing treatment.

*a) Contextual triggers*. Even when participants received what they saw as outstanding medical care in the hospital, they were reminded of their poor treatment in jail. They described how the sight of the healthcare providers and the equipment caused anxiety. In the context of treatment, they identified a variety of factors that served as stressors and induced feelings of panic. They mentioned needles, belts, and straps, along with the odor of old buildings and scissors. They also noted aging walls and unpainted concrete walls, reminding them of a prison and causing difficulty breathing and a strong urge to run. They explained that just being in the operation room was quite stressful, evoking intense memories and when entering the operating room, and they stated experiences of anxiety attacks generally in hospitals. They described that some of the twires and pipes made them worry and caused reactions, such as trembling and fear.

*Antonio*: “*All those wires*, *pipes*, *and equipment*, *you know… reminded me of torture*.*”*

*Ramin*: “*Entering the operation room gave me the same feeling as entering the torture chamber*.

Participants detailed their experiences of feeling numb in a body area as a result of local anesthetic and compared it to being tortured and losing feelings in their arms while being hung upside down. Following treatment, darkness and loneliness were compared to a jail cell, eliciting strong reactions. They struggled with intense emotions for months after treatment.

*Jamil*: “*Black panic*! *The only way to manage this was to get out*. *I wish I had light*. *When I came out*, *the shop had lights*. *I walked for four hours*. *Back home*, *I could not stand the darkness*. *I couldn’t be alone*.*”*

Some participants described the digitalization of information in healthcare facilities as psychological torture, creating feelings of power and control loss, which were compared to the feelings experienced while being tortured and imprisoned.

*Jamil*: “*Maybe they (health institution) sent the message electronically*. *Where should you complain*? *Who are you going to complain to*? *In this way*, *they destroy people’s health*. *It is mental torture*! *They torture people*!*”*

The time spent waiting prior to and during treatment was recognized as a major source of stress, anxiety, and dissatisfaction. The participants remarked that waiting for assistance reminded them of the lack of accountability they encountered in their own country’s political regime and in prison.

*Jamil*: “*They (healthcare providers) won’t help*! *How can you wait*? *How do I explain this*? *Sometimes*, *I sit and think about the regime*. *No responsibility to the people*!*”*

Participants stated that these feelings connected to waiting time were persistent and challenging to manage.

*Tabarak*: “*It’s a feeling that doesn’t go away*, *that doesn’t go away by itself*. *It reminds you of things that you have gone through*, *and it does not disappear by itself*.*”*

One of the participants stated that when he was hospitalized, the screams of other patients, particularly women, bothered him greatly because they reminded him of women tortured while he was in prison. He explained that all of the memories of the torture returned, and that such episodes made him sick for several days or weeks.

*b) Lack of control as a trigger*. The participants revealed difficulty in accepting situations in which they lost control during treatment. Despite their difficulties with the treatment’s unpredictability, the participants appeared to have developed their own control-maintenance strategies. For instance, refusing general anesthesia before surgery because of fear. As a result of his complete lack of control during general anesthesia, Jose expressed feelings of vulnerability. He also compared general anesthesia drugs to those used in torture.

*Jose*: “*When I get into the operating room*, *I don’t think about what I experienced*, *but then they try to give me drugs and so*…*”*

Participants reported feelings of loss of control during treatment and stated that the body reacts automatically to stimuli, needles, medications, and equipment. These reactions simply manifest themselves in unexpected ways. They also described communication issues with healthcare providers as difficult, particularly when interpreters were needed but not available. Participants described feelings of dissatisfaction and powerlessness. These emotions reminded them of the imbalance in the power dynamic between them and the torturers. Other participants were concerned that interpreters had connections to their home country’s government and were stressed by the presence of interpreters.

*c) Pain as a trigger*. The participants provided specific examples of the limitations they faced as a result of torture and treatment-related pain. They discussed their experiences receiving care in surgical departments by recalling a distressing situation in which the assistance of healthcare professionals was vital, particularly in the absence of a social network. They described the pain they experienced after waking up after surgery in the ICU as excruciating, which drove them to act out, necessitating that hospital professionals restrained them. They said that the torture made them stronger, allowing them to endure medical care. Nonetheless, the excruciating pain following surgery prompted feelings of hopelessness and helplessness, which served as a reminder of torture and a barrier to forgetting it.

*Bashir*: “*The pain reminded me of what I try to forget*.*”*

One participant described unbearable pain that escalated and became intolerable as a result of treatment, eliciting feelings of desperation. He noted that he frequently cried and was uncertain what to do. Another participant described the pain he endured during a rectal examination as excruciating because he had not been given anesthesia or medication beforehand. After surgery, he was unable to sit for several days. This brought back memories and feelings of torture.

*Tabarak*: “*The pain*, *it was not a good feeling*. *Because then I think of torture*.*”*

The participant stated having difficulty coping with terrible memories linked to torture and translating these emotions into pain associated with treatment. When he resumed his typical habits following treatment, this was also reported as giving him the desire to avoid some activities and future treatment.

In addition to the pain caused by surgical procedures, the majority of participants described how certain things or situations that reminded them of previous traumatic experiences could trigger involuntary, unpleasant physical or mental responses. As described by Tiam, reactions are “automatic,” particularly in situations involving an unexpected element. He described how he was reminded of unanticipated incidents of torture whenever the treatment involved any unexpected aspect.

**Fig 2:**
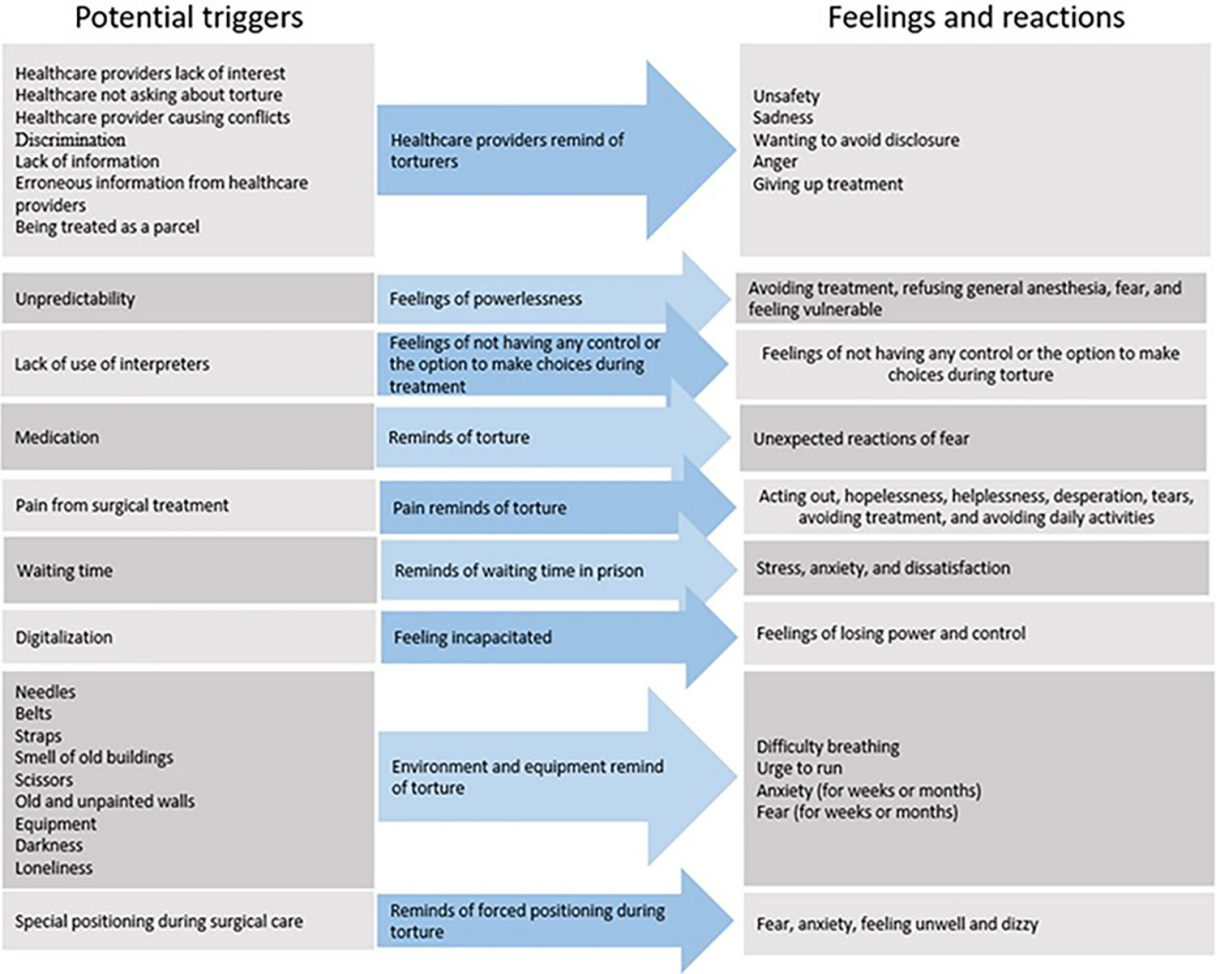
Summary of the presented potential triggers, feelings, and reactions caused by the triggers.

Fig 2 depicts the above-mentioned potential triggers, as well as the feelings and responses they induce during treatment.

#### 4. Avoidance

This theme addresses the participants’ strategies to attempt to minimize or neutralize the perceived threats, dangers, or anxieties related to treatment.

*a) Avoiding healthcare*. Regardless of their beliefs about their ability to deal with obstacles encountered during consultations and treatment, all the participants compared their prior and current conditions, indicating a decrease in seeking healthcare. One participant mentioned that his unpleasant experiences related to shoulder surgery rendered him unwilling to seek healthcare services again.

*Bashir*: “*When my heart was operated on*, *it was fine*. *But*, *in connection with the shoulder*, *it was very dramatic*. *I no longer want to visit the healthcare system*.*”*

This participant described lack of information, the absence of interpreters, and lack of control over his own treatment as factors that made him feel like he was being subjected to torture. Another participant described losing motivation to seek healthcare again because of pain during and after a rectal bleeding examination. These experiences seemingly caused him experiencing torture again, with strong and negative feelings lasting for days or weeks after treatment. He expressed disappointment in having to return to the hospital.

*b) Avoiding disclosure*. The participants reported their avoidance of disclosure during clinical encounters and provided their rationales and beliefs for doing so. One of the reasons they feared disclosing their torture experiences was their inability to anticipate responses from healthcare providers. The participants said that they did not disclose their torture experiences at the hospital because they believed that these experiences were unimportant to the healthcare staff. However, they would have appreciated being asked about their history. Some participants reported obtaining proper care; nevertheless, they never mentioned their torture experiences to healthcare practitioners since they were never asked. If the healthcare workers had asked them about their torture experiences, the majority would have told them. One participant stated that he never initiated a conversation with healthcare providers to discuss his torture experiences out of regard for healthcare providers.

*Tiam*: “*I am not sure if they can bear to hear such a story*.*”*

Participants viewed the fact that healthcare providers never asked about their past as an unpleasant experience, making them feel unimportant, similar to how they experienced during torture and incarceration.

*Tabarak*: “*It‘s not good not to be asked to get to know me better*. *It would have been nice because then one can*, *in a way*, *understand what I have gone through*.*”*

#### 5. Suggestions for healthcare providers

This theme includes suggestions made by participants to improve care quality and prevent re-traumatization.

*a) Communication between different organizations*. Participants seemed to realize that lack of communication between their general practitioner and specialist health service contributed to the perceived poor quality of healthcare. Tiam proposed that GPs report the patient’s experiences of torture to the hospital. Due to lack of documentation in healthcare, participants reported feeling frustrated.

*b) Asking about torture*. All participants agreed that anesthesiologists and other healthcare professionals should question patients’ backgrounds, particularly about torture, because it is difficult for patients to voluntarily provide such information. Some participants reported that they did not share their torture experiences with medical professionals because they deemed the information useless. According to them, medical professionals should inquire. Participants concurred that direct, pertinent questions were preferred. They also emphasized the significance of asking patients about their backgrounds with candor and genuine interest.

One of the participants expressed disappointment that no one inquired about his past while he was hospitalized; this was his only criticism regarding the care. Given the risks of his heart surgery, he expressed surprise at the lack of questions regarding his torture experiences.

*Antonio*: “*It was surprising that no one asked about my background*, *given the widespread illness and degradation that followed*.*”*

He noted that when he opted on his own to discuss torture with healthcare professionals, he received attitudes displaying a lack of education on the topic.

*Antonio*: “*Finally*, *when I explained it to someone*, *they did not comprehend what it was*.*”*

*c) Adjustments*. The participants suggested longer-term monitoring after treatment, and they were dissatisfied with ambulatory surgery and spending the night at home after surgery. Following surgery, the participants suggested that patients who had been tortured spend the night in the hospital. They mentioned that healthcare professionals should assist patients who were having difficulty filling out forms and recommended that digitalization be adjusted for those with low levels of education, those who have no one else to ask, and elderly patients. In addition, it was suggested that patients who had been tortured receive sedatives prior to surgery. Despite it being impossible to completely eliminate tension, he stated that it was possible to moderate it. Thus, it was possible to downregulate the degree to which patients reexperience torture-related thoughts and emotions before surgical treatment.

Based on his own positive experiences, one participant suggested that patients be given the option of listening to music while undergoing surgery under local anesthesia.

*d) Being seen by healthcare providers*. The participants expressed a desire “to be seen and acknowledged” by healthcare professionals, stating that doing so would facilitate the formation of a therapeutic relationship. In addition, the participants’ perspectives on the unsuccessful approaches of health professionals responding to their healthcare needs were highlighted. They emphasized the importance of healthcare providers “seeing” patients as the unique individuals that they are and paying closer attention to them. They also suggested that healthcare providers should consider a torture history when treating patients.

A sense of humor was suggested as a strategy to help the patient relax. At the same time, it was considered critical to take patients seriously. Positive emotions, such as satisfaction and happiness, were described as a result of healthcare personnel knowing and using the patients’ names during treatment. Being kind as a healthcare provider, according to the participants, made them feel safe, whereas medication alone was ineffective. This may be combined with other competencies required of healthcare professionals.

*Jamil*: “*A human feeling*. *You become a nurse or a doctor; it is different from a grocery store or a bank*. *You have a responsibility*!*”*

*e) Use of interpreters*. Participants described their negative reactions to the general lack of use of interpreters, particularly the lack of qualified interpreters. However, the main message was to encourage healthcare providers to use qualified interpreters. When the patient and healthcare providers did not speak the same language, the participants stressed the importance of using interpreters. They also shared their negative experiences during consultations, treatments, and a hospital stay that did not include an interpreter. When it comes to treating torture survivors, the first piece of advice they gave was to use professional interpreters.

*Bashir*: “*First*, *someone like me who doesn’t know the language*… *it’s important to use an interpreter*!*”*

*f) Competence and knowledge of healthcare providers*. As a result of their negative experiences with surgical care, the participants proposed that healthcare providers be aware of torture methods to provide better healthcare to torture survivors. Participants claimed that healthcare providers should be aware of the needs of torture survivors in relation to the consequences of torture. It was also suggested that rather than waiting for patients to request it, healthcare workers should know when to refer them to a psychologist, and several participants questioned the health professionals’ ability to understand their torture-related health challenges. The participants agreed that healthcare providers should be aware of how surgical treatment could interfere with the feelings and thoughts of torture survivors and should take the initiative to refer them to a psychologist following surgery.

## Discussion

This qualitative study investigated the experiences faced by torture survivors undergoing surgical examination and treatment and analyzed the trauma-related reactions experienced by at various stages of surgical treatment. Previous research [2] has mentioned difficulties torture survivors may face when undergoing surgical treatment, but to our knowledge, this is the first in-depth examination of how survivors experience undergoing surgery, with a focus on re-traumatization.

The participants described a variety of distinct aspects that can lead to re-traumatization during treatment. They considered the relationship with healthcare providers, the atmosphere, equipment, sounds, and waiting time. This is in line with other research on somatic treatment for torture survivors [5]. During the interview, participants spent a significant amount of time discussing their thoughts and experiences with medical professionals’ attitudes. Because of the relational aspects of torture, it is important to note that the relational aspect of the treatment causing remarks and responses to health professionals’ attitudes are critical to the re-traumatization process. Because torture is intended to destroy a person and his relationships with others, survivors have difficulty having a relationship and communicating with medical personnel [7]. As a result, a significant portion of the responses related to contextual aspects may have relational roots, and the solution to dealing with these triggers is within the relational aspect of clinical encounters [7]. The participants highlight different aspects of clinical encounters that demonstrate an additional preoperative anxiety and a reduced distress tolerance, which is consistent with the findings of other studies focusing on the vulnerability of torture survivors [63, 64]. This may also be because symptoms are more pronounced in torture survivors who are refugees than in those who remain in their homelands. This is likely due to the additional stress that comes along with having to adjust to a new culture while also grieving the loss of one’s family, community, and country [65].

According to the accounts of the study’s participants, little, or no effort has been made by healthcare providers to identify tortured patients in the surgical course. This is problematic because important aspects of patients’ health are ignored, putting them at risk of receiving substandard care. This is consistent with the findings of a Danish study [34] including 300 migrants, in which only about half of the torture survivors were asked about their torture history by their primary care physician. The authors [34] of the study recommended a more systematic approach to identifying torture survivors in clinical settings as well as a better understanding of when and how to ask. This recommendation is supported by the study’s findings regarding the lack of identification of torture survivors in healthcare, and it suggests the development of an active method for measuring survivors. Ideally, triage would have occurred before the surgical procedure began. The lack of tools for identifying torture survivors should also be addressed in future research [34].

This study’s participants discussed their experiences in terms of difficulties in receiving surgical treatment and acknowledged that the need for surgical treatments placed them in a disadvantageous position with few options, forcing them to accept situations, interactions, and a context that induced stress and fear reactions by evoking memories of their torture experiences. When participants described aspects of torture, they also alluded to a number of potential sources of triggers. Hunger, for example, was described as difficult to manage during torture, suggesting that fasting prior to surgical treatment may trigger feelings associated with torture, affecting the patient’s general mental state even before treatment. The participants describe various aspects of clinical encounters that demonstrate increased preoperative anxiety and decreased distress tolerance, as described in other studies [63, 64]. Additional stress, comes along with having to adjust to a new culture while also grieving the loss of one’s family, community, and country [65].

One of the most problematic issues identified in the participants’ experiences was the invasiveness of the treatment, which triggered trauma-related reactions. This is consistent with other studies’ findings [2, 5, 66]. The invasive nature of surgical treatment may raise concerns about whether torture survivors require any kind of preparation for surgical treatment; for instance, psychological support should be provided before and after surgery to avoid re-traumatization. Measures of this type are justified by the fact that torture survivors have unique needs, which have been documented in previous studies [23].

According to our findings, the body’s experience of pain is defined as an agent in the process of re-experiencing torture trauma during treatment. Similar findings were published by Taylor et al. (2013), who described how pain can evoke memories of trauma and how these memories affect pain perception [67]. This description may explain why memories of torture trauma may intensify the pain of surgical treatment, suggesting that torture survivors may require pain relief treatment during and after—which differs from standard pain management. Throughout the interviews, the participants described powerlessness in relation to pain related to treatment suggesting that they find it more difficult to endure suffering in clinical settings than in other settings. This is related to the patient’s association of the treatment settings with the torture settings, and the presence of healthcare practitioners and the power they represent. The presented aspect may attribute an authority element to the pain, causing the surgical pain to increase. This dynamic may reflect power dynamics in torture, as discussed by Pérez- Sales (2019), who described the consequences of healthcare providers being involved in torture and how this creates a symbolism in which uniformed healthcare providers become a representation of torture perpetrators and executing authorities [68]. According to this analysis, healthcare practitioners appear to be an extension of the power that might trigger pain and memories of torture; as a result, clinical modifications may be required to mitigate and balance the power dynamics between healthcare providers and patients who have experienced torture. The participants described healthcare personnel and their attitudes as catalysts for the reactivation of feelings linked to torture experiences. This requires close attention to how healthcare practitioners behave as well as how their thoughts and emotions influence their views. The mentioned underlines the need to invest in healthcare practitioners’ education about the impact of their attitudes on the treatment of traumatized survivors. This is also outlined in the quality standards presented by Cohen and Green (2022), who underlined the significance of the practice of the principles of trauma-informed care in preventing the re-traumatization of torture survivors throughout treatment [69].

Torture alters a person’s survival strategies, replacing faith in mankind with despair and loathing [23]. This was echoed in our study in which participants reported having trouble feeling confident in healthcare providers. The findings were consistent with those of a study of torture survivors in Finland and Sweden, which revealed significantly lower levels of confidence and trust in authorities and public service providers compared with refugees who had not been subjected to torture [70]. Some torture survivors appear to function in a relatively normal manner for years, with relatively latent symptoms of PTSD, and a surgical procedure may activate these symptoms. Re-traumatization may result in functional loss or diminution of skills that are not necessarily dissociative in nature, but rather involve substitute actions for higher level skills. In the aftermath of a re-traumatic experience during treatment, the survivor may lose the ability to regulate affect, e.g., impolite or aggressive behavior towards healthcare personnel, or develop feelings of losing social support during treatment because relationships with healthcare providers become threatening or too taxing for the survivor’s affected mental level. This suggests that treatment should first focus on facilitating a gradual attachment with the provider as a prerequisite to increasing levels of trust [42, 43, 71]. As difficulties in establishing relationships [7] and a lack of trust in healthcare providers can increase the risk of re-traumatization, it is crucial to create trust in the provider–patient relationship in order to prevent re-traumatization [7]. For this, mutual respect must be built by responding to patients in a trauma-informed manner and protecting their privacy in the physical examination and in the medical record by obtaining consent throughout the process. It is crucially important to ask and tell a patient when you need to touch them and why, maintain eye contact, and explain and request input on the plan of care [1]. While much of this may seem intuitive, these practices have been shown to be lacking in the many healthcare interactions described by the participants in this study. Being recognized as a torture survivor in a clinical context may empower patients and foster a trusting patient-provider relationship. This balance appears to be achieved automatically in the participants who work as healthcare professionals.

Despite the fact that some torture survivors are more vulnerable than others, some torture survivors never develop any sort of mental issues and recover from the trauma without treatment [17, 28]. The resilience, transformation, and recovery in these patients, as opposed to what others may perceive as their weakness and their pathological behavior, must receive significantly more focus from healthcare providers in order to meet the survivors’ needs. The ability to cope with the aftermath of trauma and possible re-traumatization during treatment can be supported by healthcare providers by searching for ways to numb the pain and if healthcare providers pay attention to what is important to the patient in terms of their resilience [28, 72].

In this study, participants also reported encountering strong skepticism from healthcare providers, particularly when presenting symptoms of diffuse pain. This dynamic is comparable to that of torturers who do not believe their offers. The idea that healthcare providers do not believe in a patient’s symptoms may resemble the dynamic under torture, discouraging patients from disclosing their torture experiences and impeding or preventing the formation of trust [43]. Accept and validation may support the therapeutic value of making sense of one’s own torture experiences as well as the human need to develop a sense of truth [42]. According to Steger and Park (2012), making sense of what happened is one of the most important strategies for trauma recovery. As a result, healthcare providers should meet the survivors as open-minded and compassionate witnesses adopting a moral and solidaristic attitude towards the survivors [73, 74]. To accomplish this, healthcare professionals may require additional training programs and experience [75] and conducting additional research into the types of educational programs that can help to improve the quality of treatment of torture survivors and prevent re-traumatization should be a priority.

Several participants in this study revealed that their torture experiences left them with feelings of shame, which became reinforced during treatment. These feelings of shame reinforced their willingness to endure painful treatment and to not lose their control, and their dignity which tch is fundamental to feel secure. According to Dalgaard et al. (2021), survivors of torture frequently feel shame as a result of their experiences, and many survivors are afraid to disclose their experiences, even in the context of treatment [76]. Dalgaard et al. (2021) stated that it is essential for healthcare providers who work with torture survivors to comprehend the nature of traumatic experiences in order to ask appropriate questions, even when survivors have difficulty disclosing their torture experiences and sequelae [76]. Contrary to this suggestion, some participants reported encounters with healthcare providers who were uninterested in asking about the patient’s prior experiences or listening when the patient attempted to speak. It is possible that patients misinterpret the silence of healthcare providers as a lack of interest when, in reality, they lack the courage to inquire because they do not know how to respond to the disclosure of a history of torture. This was also described by Amris et al. (2019), along with a recommendation to ask patients about torture [36]. According to Amris et al., when a history of torture is not disclosed, there is a possibility that healthcare providers will make erroneous clinical decisions. As a result, inadequate or even inappropriate treatment may be administered, leading to unfavorable results and re-traumatization. Therefore, it is crucial to establish good relationships with patients and to inquire openly about any history of torture [36]. Furthermore, by bearing witness to the patient’s trauma history, healthcare providers lay the groundwork for safety and trust [42]. Healthcare professionals must also be able to handle patients’ histories if they choose to tell them. In a study on dental care for torture survivors, Høyvik & Woldstad offered advice on how to respond and what to say when patients reveal their torture experiences [77]. They advised them to act with compassion and understanding. A study by Shannon involving 111 refugees from Burma, Bhutan, Somalia, and Ethiopia collected data through interviews and 13 focus groups regarding how refugees believed healthcare providers should inquire about their prior experiences [41]. The advice given by the participants focused on asking direct questions, putting the patient at ease, and using trained interpreters [41]. This is consistent with what the participants in our study suggested. Asking about the patient’s past may also provide an opportunity for them to express their needs, as traumatized patients typically have difficulty communicating directly and openly regarding their needs, feelings, and perceptions [78].

Some participants described the discriminatory and authoritarian attitudes of healthcare providers as difficult to deal with, evoking sensations comparable to those experienced during torture. According to a study by Garoff et al. (2021) that included 162 cases of torture, torture survivors reported greater discrimination by authorities and in daily life compared with survivors of other trauma and non-traumatized migrants. The authors also hypothesized that this may be because survivors of torture have become more sensitive to perceived injustices [70]. Another study examined the effects of discrimination in inpatient care, highlighting how negative interactions with providers frequently caused re-traumatization in marginalized patients [79]. According to Gutowski et al. (2022), departments should assess racial trauma by inquiring about prior discrimination experiences and should implement trauma-informed care principles to prevent discrimination in healthcare [79]. Because discrimination is frequently associated with traumatic stress, it is important to be aware of this issue when caring for torture survivors [80].

This study’s findings indicate that what can trigger torture-related emotions and reactions during surgical treatment is partially related to the type of torture method to which the patients were subjected. This means that health professionals must be aware and have knowledge of these methods in order to make adjustments to prevent re-traumatization. Several triggers, including lack of control, pain, and environmental factors, were reported by our study participants as contributing to the re-experiencing of torture trauma. As stated by Høyvik et al. (2021), it is essential for healthcare providers to be aware of and knowledgeable about the various triggers as well as the need to investigate patients and triggers individually [5].

Participants in our study revealed that different triggers elicited distinct responses, and that the same trigger could elicit distinct responses in different contexts and at different times. These intense reactions may indicate re-traumatization, with feelings and even symptoms persisting for hours, days, or weeks after treatment. To reduce the impact of triggers in general, healthcare providers need knowledge about what can re-traumatize and implement adjustments to neutralize the triggers related to the environment, such as the equipment and instruments used during treatment. Preventing re-traumatization during treatment is crucial because, after re-traumatization, patients must once again organize their lives around their trauma histories and the intense emotions and reactions they experience while receiving treatment. It may be difficult for the patient to manage this process of transformation on their own, as it may require effort and a high level of resilience. Typically, healthcare providers do not observe this process because patients are sent home immediately following surgery. Recuperation occurs at home without supervision and can present difficulties for the patient. After retraumatization, the process of recovery can be lengthy, and patients may struggle to manage life for an extended time [48]. Further research should therefore focus on the transition between treatment in hospitals and recovery when the patient is discharged.

It is crucial that adjustments to prevent re-traumatization during treatment are not dependent on who is treating the patient, but rather are routinely implemented. For this, it is necessary to develop guidelines, including the necessary adaptations to prevent re-traumatization. Concurrently, it is essential to raise healthcare providers’ awareness of the fact that torture survivors’ responses to treatment are heavily influenced by their interactions with medical personnel [7], and that a supportive attitude on the part of healthcare providers can be the key to preventing re-traumatization. Additionally, healthcare professionals may recognize the symptoms of re-traumatization and how the patient is transformed into the process. By understanding this transformation process as typical stress response, healthcare professionals can recognize retraumatization and assist patients with recovery. Some of the participants in this study reported reactions that are in conformity with a “fight” reaction and also submission reactions during treatment. This echoes findings in a case study with a torture survivor receiving surgical treatment and reacting with flashbacks and aggressivity after anesthesia [10]. According to Crosby (2007), after receiving general anesthesia, survivors of torture may experience flashbacks and the identification of flashbacks enables prompt therapeutic intervention. Typically, the flashbacks of torture survivors involve their torture, and providers should begin with the management of flashback triggers. The patient may be reassured that they are safe in the hospital and not in a dangerous situation from the past when they are informed of the time and location. This is often more effective and safer than pharmacological interventions that may exacerbate symptoms [10]. Crosby (2007) suggests that torture survivors with a known history of PTSD would benefit from having a familiar person or caregiver present upon emergence to support transformation and recovery. A familiar person can understand a patient’s preferred recovery strategies. It is also essential for healthcare providers to be aware of the protective factors that may aid a patient’s recovery after re-traumatization. According to Kira (2014) in the study about Arab refugees, family and religion are crucial recuperation factors [81].

It is crucial to recognize dissociative responses to stress when re-traumatization occurs. As dissociative traumatic memories are common following torture [82], it is essential to determine if the trigger causing the patient to re-experience trauma symptoms related to torture also triggered dissociative traumatic memories related to treatment. Some of the participants in this study reported not remembering the intense reactions they experienced upon awakening from general anesthesia, despite being informed by healthcare professionals. This could be a sign of dissociative amnesia as psychological defense [82, 83] and consequently, a sign indicating that the patient requires further psychological treatment.

As stated previously, we are discussing attitudes toward healthcare and communication skills. The majority of patients cited communication difficulties, issues with information delivery, and a lack of interpreters as treatment barriers and causes of re-traumatization. Other studies focusing on the use of interpreters echoed findings in this study, and reported the risk of re-traumatizing patients due to a lack of interpreters [84] and recommended the use of interpreters even when survivors appeared fluent during the initial meeting. Furthermore, interpreters must be trained and have prior experience working with torture survivors. This supports this study’s participants’ claims that they were dissatisfied with the interpreters’ competence. Some participants were dissatisfied with the use of interpreters, expressing fear and associating interpreters with authorities from their home countries. Due to its anonymity, Williams and Hughes (2020) proposed phone interpreting as a solution to such problems [84]. In addition, they suggested that interpreters be briefed and debriefed. If the survivor has specific requirements regarding gender, ethnicity, or preferred language, these preferences should be honored [84].

Another trigger mentioned by a participant worthy of discussion was witnessing the pain of others in a clinical setting. Torture victims are frequently forced to hear or see others tortured in prison, and they may also be forced to torture others [85]. This can have a greater impact on people who live in collectivist societies, where a stronger sense of shared identity with other members of their society or family makes other people’s suffering one’s own [86]. Thus, elements related to the treatment of others, or simply being present when others receive inappropriate treatment, can elicit the same strong reactions as one’s own treatment experiences. This dynamic was also discussed by Oaterholtz, who explained that witnessing the pain of someone else is more psychologically damaging than experiencing pain oneself [87].

The perception of the passage of waiting time was another trigger discussed by the participants. They all felt that the waiting time was extremely long, and it was of great significance to them, even more important than the treatment itself. When a patient awaits therapy, nothing occurs, which may be analogous to the situation in prison shortly before dreadful things occur. Thus, we hypothesized that the participants attempted to equate clinical waiting time with the period preceding torture. In this instance, it appears that the participant was unable to trivialize the waiting period, instead elevating it to a crucial feature of the therapeutic situation.

The reactions to various triggers described in this study indicated that re-experiencing trauma due to associations between surgical treatment and torture caused similar feelings to those experienced during torture, including anger, frustration, fear, shame, and despair. These feelings, which are typically accompanied by reactions such as loss of control, breathing difficulties, stress, screaming, and acting out, are referred to as the “fight-or-flight”response because they serve as a survival mechanism, allowing individuals to react swiftly to life-threatening situations [88]. Similar reactions were described by Høyvik et al. (2021) in their study of torture survivors who received dental care. According to Høyvik et al., the factor of unpredictability was significant and affected how torture survivors responded to treatment [5]. Some of our study participants expressed intense anger in response to unpredictability and a lack of information during treatment as well as unexpected actions by healthcare providers. This placed them in previously described disempowerment situations, evoking the feelings of powerlessness felt by torture victims whose tormentors ignored their individual needs. These feelings could lead to avoidant behavior, causing torture survivors to interrupt or discontinue treatment. This was also described by our study participants, who reported rage, insomnia, and nightmares following treatment as well as a desire to never seek medical attention again. According to Adenauer et al., avoidant behavior is a component of the “fight-or-flight”response [88]. Such responses are typically characterized by (i) reminders of the exposure (including flashbacks, intrusive thoughts, and nightmares); (ii) activation (including hyperarousal, insomnia, agitation, irritability, impulsivity, and anger); and (iii) deactivation. These reactions can make it challenging for torture survivors to seek medical care, resulting in long-term impairment in health [88, 89].

Our participants described attempting to forget and move on from their hospitalization after being discharged. Despite this, it appeared that general anesthesia-induced memory gaps may have caused stress and associations with a lack of memories during torture when the individual fainted. One of the typical responses to trauma is fainting [45], which not only indicates that the situation may be causing stress but can also indicate that the individual has been re-traumatized. It is imperative that healthcare providers pay close attention to reactions of this nature and determine if additional procedures are necessary.

To bridge this gap, healthcare providers may need to speak with the patient prior to hospital discharge. When both experiences (torture and care) are associated with negative connotations, the desire to forget torture is analogous to the desire to forget to receive care. A number of participants expressed the desire to forget treatment-related events, which could be attributed to the distress caused by care-related events that motivated avoidant behavior. This was also highlighted in a study on pain treatment for torture survivors, which discovered that negative treatment experiences reduced treatment adherence [39].

Due to the potential for surgical procedures to re-traumatize torture survivors [2], healthcare professionals working in surgical departments should be familiar with trauma-informed care principles, as suggested by Grossman (2021) [1] in order to prevent re-traumatization. This study demonstrated the critical importance of implementing a trauma-informed response throughout the institution. Grossman provided recommendations for implementing this type of care to prevent re-traumatization. The study contains examples from healthcare settings to illustrate how organizations are advancing this patient-centered, trauma-informed approach to care. One of the suggested strategies is to educate healthcare professionals about the effects of trauma on patients and the necessary adjustments to prevent re-traumatization [1]. Adapting the institution as a whole is what benefits patients [1]. Consequently, developing and implementing guidelines to prevent re-traumatization during surgical treatment can be a priority and the first step in implementing trauma-informed care to prevent the re-traumatization of torture survivors during surgical care.

Healthcare providers working in surgical departments see patients who are bloodied, swollen, and broken, and although it may appear that these are the most dramatic injuries, this is not always the case. Unfortunately, this does not tell the whole story for many patients, as lifelong exposure to multiple traumatic events is regrettably common [90] and among the patients they meet some are survivors of torture [2]. Surgical care professionals are familiar with the concept of psychological trauma, but adapting surgical treatment to traumatized patients is still a challenge [2, 90]. Healthcare providers working in surgical departments have a unique opportunity to embrace trauma-informed care, develop innovative solutions, and advocate for universal implementation to meet the torture survivors special needs but they must be educated on these principles as well on the methods and consequences of torture [2, 46]. The fact that different professionals may receive different training in treating torture survivors and may lack a common theoretical approach to solve the practical problems that arise when treating torture survivors is an additional barrier that prevents healthcare providers working in surgical departments from providing adequate care to torture survivors. As a result of the management of healthcare institutions expecting high levels of productivity, healthcare providers are also subjected to stress in the workplace. This can lead to the development of unhealthy routines, such as spending less time with patients. This suggests that awareness about the necessity of providing specialized care to patient groups such as survivors of torture should be implemented from the highest levels of healthcare institutions [78].

## Limitations

This study does not seek to generalize its findings. The participants we were able to recruit for the study were open and willing to share their experiences, whereas many others who did not wish to participate in the research might have had different interactions with health services. The study’s sample was relatively small due to the characteristics of the community and the study site’s low patient volume. However, this was taken into account when designing the study, and numerous reliable data sources helped to increase the validity of the results [91]. Compared to other qualitative studies involving torture survivors [5, 39], our sample of eight informants exhibited a satisfactory range with respect to their country of origin, type of torture, and surgical treatment. Despite an extensive recruitment strategy [50], we failed to recruit women for the study. This is a significant flaw, as women may have different healthcare needs and different ways of coping with trauma [92, 93]. However, our findings underpin the findings from a previous synthesis of qualitative studies that included both men and women [49], indicating that our results are relevant for both sexes. Despite the small number of participants, a wide range of perspectives and experiences were shared, covering a wide range of issues.

The disadvantages of using interpreters were mitigated by hiring interpreters with professional training and extensive experience as well as by conducting interviews through interviewers who had prior experience communicating through interpreters. However, using non-native languages in the study interviews may have impeded the expression of certain issues. Participants who declined interpretation assistance, on the other hand, spoke Norwegian fluently. This provides benefits that are lost when interpreters are used because it prevents participants from freely expressing themselves.

Although the presence of therapists during two of the interviews and wives during two of the interviews could have induced bias, this was counterbalanced by the possibility that it made the participants feel more at ease, secure, and confident.

The interviewer’s extensive familiarity with the subfield of the healthcare context that served as the study’s setting is one of the project’s many strengths. In this study, both the participants and the researchers brought their own conceptual maps of the phenomena to the meeting, promoting reflexivity. New maps were discussed, negotiated, and developed throughout the interaction in order to generate new knowledge and understanding of the phenomena [94]. Multiple researchers analyzed the data to ensure the fairness of the procedures and to neutralize any potential bias.

## Conclusions and implications for practice

The present study adds new aspects to our understanding of the re-traumatization of torture survivors undergoing surgical treatment. It is essential for healthcare professionals to understand the significance of the relational aspect of torture and how this affects the relationship between survivors and healthcare professionals. Further, healthcare providers must identify patients who may be torture survivors and be conversant with the challenges they face. Therefore, it is necessary to develop tools that can assist healthcare providers with this task. The reactivation or worsening of trauma-related reactions was cited as a significant consequence in our discussion of the factors that make it difficult for torture survivors to undergo surgical treatments. We presented healthcare providers’ attitudes, lack of trust, lack of knowledge, surgical procedures, general anesthesia, environment, lack of control, and pain as re-traumatization contributing variables. Healthcare providers who interact with torture victims should be aware that these patients frequently suffer from life-altering health issues, which complicate the clinical picture and considerations of patient trust. Although surgical care may be necessary, associations between the surgical treatment and the patient’s prior torture experiences may provoke physical and psychological responses as part of the body’s stress response, resulting in problems with heart rate and respiration, as well as aggression, anxiety, and fear after surgery. Due to the importance of preventing such reactions during treatment, it is necessary to enhance healthcare providers’ understanding of torture, the difficulties faced by torture survivors undergoing surgical treatment, and to modify clinical practice. Appropriate time must be allocated to ensure quality care, and medical personnel must ask patients about torture experiences. Trauma reactions are influenced by diverse stimuli based on the individual’s experiences, and what causes intense reactions during treatment must be investigated on an individual basis. Creating predictability, as well as providing patients with understandable information, is essential. This may necessitate using professional interpreters. Healthcare workers should learn how to listen to patients’ accounts of torture and support them throughout the disclosure process. Another critical skill is the ability to assist patients when severe reactions occur during surgical treatment. Important aspects include the knowledge and ability to recognize and comprehend the transformation process into re-traumatization, as well as the understanding of what can promote recovery from re-traumatization. As a first step in implementing trauma-informed care to prevent the re-traumatization of torture survivors during surgical treatment, the development and implementation of guidelines are necessary.

## Supporting information

S1 File Interview guide. (DOCX)

S2 File Additional questionnaire. (DOCX)

S3 File Written consent–participants. (DOCX)

S4 File Written consent–relatives. (DOCX)

S5 File The protocol to assist participants in dealing with strong reactions during and after the interview. (DOCX)

S1 Table Overview of the participants, the described torture methods, context and sequels, type of treatment, treatments context, and healthcare providers involved in the treatment. (DOCX)
