# Supplementary material for: Torture survivors’ experiences of receiving surgical treatment indicating re- traumatization
Source: PLoS One. 2023 Oct 17;18(10):e0287994. doi: 10.1371/journal.pone.0287994 (PMC10581467; doi:10.1371/journal.pone.0287994)
Supplement: S1 File — (DOCX) [file pone.0287994.s001.docx]

**Supplemental file S1**

**THE INTERVIEW GUIDE**

Preparing for the interview

Ahead of the interview, we will do everything possible to ensure that the participants feel safe by demonstrating utmost respect and an appreciation for the fact that they may need to protect themselves during the interview. We will do everything possible to give the participants the opportunity to make their own decisions so that they feel in control during the interview. We will not speak loudly, avoid mood or attitude changes, and refrain from expressing disbelief. We will allow informants to ask questions or request clarification, and we will rephrase any questions that appear unclear or are not understood.

During the interview, the participants will have the opportunity to catch up with themselves, if necessary, and to discuss their experiences in a manner that is most comfortable for them.

After each question, the participant will be given a few seconds of silence to collect his thoughts and determine how to respond. If necessary, we will also give the participants the opportunity to take breaks.

We've prepared a set of questions that we'll use if the informants struggle or lose the thread during the interview. Torture survivors may have suffered brain damage as a result of blows to the head and other types of traumas. This can result in cognitive issues and the inability to remember specific things. Furthermore, a survivor may have an emotional memory of what happened but not remember the specifics.

In the interview, we combine open questions and more direct questions. The first questions are the most important while the last questions are used more as a clue and respondents can skip them if they wish.

The most important questions are: "Can you tell us your story?" This question is asked in two parts: Related to the subject of torture and the subject of surgical treatment. To the answers to these questions, we attach follow-up questions that usually start with: "... what you just told me was interesting. Can you tell me more about that?”

The two concluding questions are also important, and the answers to these can give us important and necessary information for the project.

| INTRODUCTION |
| --- |
| We would like to ask you about your past history and present symptoms. This information will be used to help us provide you with better medical care. However, you may find some questions upsetting. If so, please feel free not to answer. This will certainly not affect your treatment. The answer to the questions will be kept confidential. |

| In-depth interview | |
| --- | --- |
| 1. Torture history and methods | Could you please tell us about your background? What were your previous experiences before coming to Norway? |
|  | Please tell me what you think are the most hurtful or scary things that have happened to you. Please tell me when and where these things happened. |
|  | What is the worst thing that has happened to you in your current living situation (i.e., refugee camp, country of resettlement, returned from exile, etc.) if it is not one of the things listed above? Please tell me where and when these things took place. |
|  | Are there any other details you'd like to share about this topic? |
| 1. Hypersensitivity (distrust, vulnerability, powerlessness, loss of control, low self-esteem, and difficulty self-advocating) | Has your trust in other people changed as a result of the torture? Can you tell which way it is? |
|  | Do you think your experiences in your home country have made you more sensitive? In what sense? |
|  | Do you think your self-esteem has changed as a result of the torture? In what way? |
|  | Do you think the torture has made it more difficult to make decisions? |
|  | Are there other things about this subject you want to tell? |
| 1. Surgical treatment | Could you please tell me why you had surgery and where you had it done? |
|  | Did you tell your healthcare providers about your torture experiences before undergoing surgery? |
|  | Could you please explain why you did or did not talk about your torture experiences? |
|  | Can you describe your experience telling healthcare providers about torture? |
|  | Are there any other details you'd like to share about this topic? |
| 1. Hypersensitivity to threats to safety | 1-Were you concerned about your safety when seeking surgical care?  2-Did you feel confident in your ability to protect yourself from any risks associated with the treatment?  3-Did you experience any anxiety before the treatment? What was causing the stress?  4-Did you try to hide your anxiety from medical professionals? How?  5-Did your interactions with health care providers cause you anxiety?  6- Did you have trust in the medical professionals involved?  7- Did you feel in control of yourself during treatment?  8- Did you believe you had a choice while undergoing treatment?  9- Did you ever feel like you were losing control during treatment?  10- Did you experience any anxiety during the treatment?  11- Did you feel ashamed or embarrassed as a result of the treatment?  12- Did you believe you were deserving of healthcare?  13- Can you describe any other negative feelings you have while undergoing treatment?  14- Can you describe your positive feelings while undergoing treatment?  Are there any other details you'd like to share about this topic? |
| 1. Triggers | Was there anything aspects of the treatment that reminded you of the torture? |
|  | Was there anything related to the health personnel's attitudes that was uncomfortable for you under treatment and reminded you of the torture? |
|  | Was there anything in the environment during the treatment that reminded you of the torture? |
|  | Are there other things about this subject you want to tell? |
| 1. Reactions indicating re-traumatization | Can you please tell about your reactions under/after the treatment |
|  | Do you feel that your health status is worsened because of your experiences under surgical treatment?  Can you please tell in which way? |
|  | Are there other things about this subject you want to tell? |
| 1. Avoidant coping | Did your view/opinion of healthcare services and healthcare professionals change after the surgical treatment?  Can you tell me how? |
|  | Have you consulted health services after the surgical treatment? Can you tell why? |
|  | Are there other things about this subject you want to tell? |
| Final Questions | What is the most important factor for you when contacting or receiving treatment from the health-care system? |
|  | What advice would you give to healthcare professionals about providing health care to people who have had similar experiences as you? |
