# Supplementary material for: Torture survivors’ experiences of receiving surgical treatment indicating re- traumatization
Source: PLoS One. 2023 Oct 17;18(10):e0287994. doi: 10.1371/journal.pone.0287994 (PMC10581467; doi:10.1371/journal.pone.0287994)
Supplement: S2 File — (DOCX) [file pone.0287994.s002.docx]

**Supplemental file S2**

ADDITIONAL QUESTIONNAIRE

This questionnaire will be administered at the conclusion of the interview. If the informant shows signs that this will be difficult, we will give the person the opportunity to respond at a later date.

PART 1: TRAUMA OCCURRENCES

Please indicate whether you have witnessed any of the following events:

|  | Yes | No | Choose not to answer |
| --- | --- | --- | --- |
| 1. Lack of shelter |  |  |  |
| 2. Lack of food or water |  |  |  |
| 3. Ill health without access to medical care |  |  |  |
| 4. Combat situation (e.g., shelling and grenade attacks) |  |  |  |
| 5. Beating to the body |  |  |  |
| 6. Rape |  |  |  |
| 7. Other types of sexual abuse or sexual humiliation |  |  |  |
| 8. Knifing or axing |  |  |  |
| 9. Torture, i.e., while in captivity you received deliberate and systematic infliction of physical or mental suffering |  |  |  |
| 10. Serious physical injury from combat situation or landmine |  |  |  |
| 11. Imprisonment |  |  |  |
| 12. Extortion |  |  |  |
| 13. Brainwashing |  |  |  |
| 14. Forced to hide |  |  |  |
| 15. Kidnapped |  |  |  |
| 16. Enforced isolation from others |  |  |  |
| 17. Someone was forced to betray you and place you at risk of death or injury |  |  |  |
| 18. Forced to physically harm a family member, or a friend |  |  |  |
| 19. Forced to physically harm someone who is not family or friend |  |  |  |
| 20. Forced to betray family member, or friend placing them at risk of  death or injury |  |  |  |
| 21. Forced to betray someone who is not family or friend placing them  at risk of death or injury |  |  |  |
| 22. Murder, or death due to violence, of spouse |  |  |  |
| 23. Murder, or death due to violence, of child |  |  |  |
| 24. Murder, or death due to violence, of other family member or friend |  |  |  |
| 25. Disappearance or kidnapping of spouse |  |  |  |
| 26. Disappearance or kidnapping of child |  |  |  |
| 27. Disappearance or kidnapping of other family member or friend |  |  |  |
| 28. Serious physical injury of family member or friend due torture |  |  |  |
| 29. Witness beatings to head or body |  |  |  |
| 30. Witness torture |  |  |  |
| 31. Witness killing other |  |  |  |
| 32. Witness rape or sexual abuse |  |  |  |
| 33. Another situation that was very frightening or in which you felt your life was in danger.  Specify: |  |  |  |

PART 2: HEAD INJURY

|  | Yes | No |
| --- | --- | --- |
| 1. Beatings to the head |  |  |
| 1. Suffocation or strangulation |  |  |
| 1. Near Drowning |  |  |
| 1. Other types of injury to the head |  |  |
| 1. Starvation |  |  |

PART 3: TRAUMA SYMPTOMS

The following are symptoms that people may experience after going through painful or terrifying events in their lives. Please carefully read each one and decide how much the symptoms bothered you.

|  | (1)  Not at all | (2)  A little | (3)  Quite a bit | (4)  Extremely |
| --- | --- | --- | --- | --- |
| 1. Recurrent thoughts or memories of the most hurtful or terrifying events |  |  |  |  |
| 1. Feeling as though the event is happening again |  |  |  |  |
| 1. Recurrent nightmares |  |  |  |  |
| 1. Feeling detached or withdrawn from people |  |  |  |  |
| 1. Unable to feel emotions |  |  |  |  |
| 1. Feeling jumpy, easily startled |  |  |  |  |
| 1. Difficulty concentrating |  |  |  |  |
| 1. Trouble sleeping |  |  |  |  |
| 1. Feeling on guard |  |  |  |  |
| 1. Feeling irritable or having outbursts of anger |  |  |  |  |
| 1. Avoiding activities that remind you of the traumatic or hurtful event |  |  |  |  |
| 1. Inability to remember parts of the most hurtful or traumatic events |  |  |  |  |
| 1. Less interest in daily activities |  |  |  |  |
| 1. Feeling as if you don’t have a future |  |  |  |  |
| 1. Avoiding thoughts or feelings associated with the traumatic or hurtful events |  |  |  |  |
| 1. Sudden emotional or physical reaction when reminded of the most hurtful or traumatic events |  |  |  |  |
| 1. Feeling that you have less skills than you had before |  |  |  |  |
| 1. Having difficulty dealing with new situations |  |  |  |  |
| 1. Feeling exhausted |  |  |  |  |
| 1. Bodily pain |  |  |  |  |
| 1. Troubled by physical problem(s) |  |  |  |  |
| 1. Poor memory |  |  |  |  |
| 1. Finding out or being told by other people that you have done something that you cannot remember |  |  |  |  |
| 1. Difficulty paying attention |  |  |  |  |
| 1. Feeling as if you are split into two people and one of you is watching what the other is doing |  |  |  |  |
| 1. Feeling unable to make daily plans |  |  |  |  |
| 1. Blaming yourself for things that have happened |  |  |  |  |
| 1. Feeling guilty for having survived |  |  |  |  |
| 1. Hopelessness |  |  |  |  |
| 1. Feeling ashamed of the hurtful or traumatic events that have happened to you |  |  |  |  |
| 1. Feeling that people do not understand what happened to you. |  |  |  |  |
| 1. Feeling others are hostile to you |  |  |  |  |
| 1. Feeling that you have no one to rely upon |  |  |  |  |
| 1. Feeling that someone you trusted betrayed you |  |  |  |  |
| 1. Feeling humiliated by your experiences |  |  |  |  |
| 1. Feeling no trust in others |  |  |  |  |
| 1. Feeling powerless to help others |  |  |  |  |
| 1. Spending time thinking why these events happened to you |  |  |  |  |
| 1. Feeling that you are the only one that suffered these events |  |  |  |  |
| 1. Feeling a need for revenge. |  |  |  |  |
