# Supplementary material for: Torture survivors’ experiences of receiving surgical treatment indicating re- traumatization
Source: PLoS One. 2023 Oct 17;18(10):e0287994. doi: 10.1371/journal.pone.0287994 (PMC10581467; doi:10.1371/journal.pone.0287994)
Supplement: S3 File — (DOCX) [file pone.0287994.s003.docx]

**Supplemental file S3**

Consent form for participants

DO YOU WANT TO PARTICIPATE IN THE RESEARCH PROJECT "GIVING TORTURE SURVIVORS A VOICE: DEVELOPING GUIDELINES TO PREVENT RE-TRAUMATIZATION DURING SURGICAL CARE?"

THE PURPOSE OF THE PROJECT AND WHY YOU ARE REQUESTED TO PARTICIPATE

This is an invitation to join a research project aimed at developing guidelines to prevent torture survivors from being re-traumatized during surgical treatment. You have been invited to participate in the project because you have previously been tortured or mistreated in your home country and have received treatment or healthcare in a surgical department.

WHAT MEANING DOES THE PROJECT HAVE FOR YOU?

We invite you to an interview in which you can tell us about your experiences with treatment in a surgical department, as well as your experiences in your home country and/or during the flight.

If you wish and/or require it, we can use an interpreter.

You will be given the declaration of consent after you have received both written and verbal information about the project, and we require your signature on the consent form.

In the project, we will record your demographic information (age, gender), country of origin, date of arrival in Norway, and date of surgery. In addition to recording your surgical history, we will also record the information you provide during the interview. During the interview, we will use a handbook with interview questions and conversational topics. In addition, you are allowed to discuss any additional experiences and circumstances you consider significant.

You choose the time and location of your interview.

A digital audio file will contain the interview.

We have scheduled two hours for the interview, but you decide when to conclude or if you need additional time.

Only the project manager will be aware of your identity.

POSSIBLE ADVANTAGES AND DISADVANTAGES

The benefit of participating in the experiment is that you will assist us in determining what therapeutic challenges torture-exposed patients face. This will help us build standards for how health care professionals can monitor torture survivors undergoing surgical therapy. This will allow you to express your experiences and ideas, and it will be beneficial to many people in the same circumstances as you. Your information will only be used for this project.

It is possible that talking about your experiences will make you feel uncomfortable.

You can pause whenever you want, take breaks, and you are not required to continue the interview if you do not wish to.

If you have any problems as a result of the interview, please contact research fellow and specialist nurse Ana Carla Schippert at 90971923, professor AKB (90134535) or professor EKG (90134535). (98845950).

We can talk to you about the interview and what happened, and we can put you in touch with a District psychiatry center if you need further assistance. If necessary, you will be assisted in contacting a medical facility.

PARTICIPATION IS VOLUNTARY, AND YOU MAY WITHDRAW YOUR CONSENT AT ANY TIME.

Participation in the project is completely optional. If you wish to participate, you must sign the consent form on the final page.

You may revoke your consent at any time and without explanation.

If you do not wish to participate or subsequently decide to withdraw, there will be no negative effects for you or your subsequent treatment. If you withdraw your consent, no further research on your information will be conducted.

You may also request that your health information will be destroyed or transferred within 30 days.

The right to demand destruction, deletion, or disclosure does not apply if the information or material has been anonymized or published in research articles. This access can also be restricted if the information has been analyzed or if it has been processed.

If you wish to withdraw from the project or have issues about it, you can contact the project manager (see contact information on the last page).

WHAT HAPPENS TO THE INFORMATION ABOUT YOU?

The information registered about you must only be used as specified in the project's purpose, and its use is expected to continue until 2025. Any extension of the use and storage duration requires the agreement of the Regional Ethics Committee and other pertinent authorities. You have the right to see the information registered about you, as well as the right to have any inaccuracies in that information fixed. Also, you have the right to access the security measures implemented during data processing. You can file a complaint with the Norwegian Data Protection Authority and the institution's data protection representative regarding the processing of your data.

All information will be handled without the use of names, social security numbers, or other identifying information (= coded information). A list of names connects you to your information via a code. This list is accessible only to the research fellow Ana Carla Schippert and the project manager Ann Kristina Bjørnnes. The information will be held safely in Akershus University Hospital's (Ahus) internal system for keeping research data, in accordance with the Norwegian Data Protection Authority's norms and procedures. For control purposes, your information will be stored for five years following the completion of the project.

Publishing of results is required as part of the research process in order for them to have an impact on clinical practice. The project's outcomes will be published as scholarly articles and a dissertation. Any publication must ensure that individual participants cannot be identified.

APPROVALS

The Regional Council for Medical and Healthcare Research Ethics has authorized project 227624 based on an evaluation of its research ethics.

SOURCE OF CONTACT INFORMATION

The Akershus University Hospital and project manager Ann Kristin Bjørnnes are in charge of the project's privacy. The project's home institution, OsloMet Metropolitan University, has performed and evaluated a risk analysis for the processing and storage of data.

You can contact research fellow/specialist nurse Ana Carla Schippert (90971923, Email: ana.schippert@gmail.com) or project manager/specialist nurse/first assistant professor Ann Kristin Bjørnnes (90134535, Email: anki@oslomet.no) if you have questions about the project or wish to withdraw from participation.

If you have issues concerning the project's privacy, please contact the institution's privacy representative at personvern@ahus.no.

The e-mail address for The Norwegian Data Protection Authority is [postkasse@datatilsynet.no](mailto:postkasse@datatilsynet.no).

I AGREE TO PARTICIPATE IN THE PROJECT AND TO THE USE OF MY PERSONAL DATA AS DESCRIBED.

**The participant**

Place and date……………………………………………………….

Participant's signature …………………………………………………………………………

Participant's name in printed letters ……………………………………………………………………………………………….

**Substitute consent**

As the next of kin of ……………………………………………………………………………… (Full name)

I agree that she/he can participate in the project.

Place and date…………………………………………………………………………………

Relative's signature …………………………………………………………………………………………..........

Relative's name in printed letters………………………………………………………………………………………

**The researcher**

I confirm that I have provided information about the project

Place and date……………………………………………………………………………………………

Signature………………………………………………………………………………………

Role in the project…………………………………………………………………………………………
