# Supplementary material for: Torture survivors’ experiences of receiving surgical treatment indicating re- traumatization
Source: PLoS One. 2023 Oct 17;18(10):e0287994. doi: 10.1371/journal.pone.0287994 (PMC10581467; doi:10.1371/journal.pone.0287994)
Supplement: S5 File — (DOCX) [file pone.0287994.s005.docx]

**Supplemental file S5**

**Project “Developing guidelines to prevent re-traumatization of torture survivors during surgical care”**

**Procedure for following up the participants**

To care for the participants, we will do everything in our power to help them feel safe, by demonstrating respect and understanding that they may need to use self-defense mechanisms to protect themselves during the interview, as strong emotions may cause a loss of control. We will prevent this by giving participants the option to answer questions and/or end the interview on their own accord. This is especially pertinent in relation to our standard questionnaire, which will be presented at the conclusion of the interview, as it may be perceived as being too lengthy. We have established plans for participant follow-up both during and after the interview, if discomfort, or strong reactions occur.

**The procedure applies to**: The researcher and the members of the project group

**Purpose and scope**: The purpose of the procedure is:

- To ensure good follow-up of participants in the project. To prevent discomfort in connection with the interview.
- To safeguard the participant's dignity and integrity throughout the interview,
- to ensure that the project process is in accordance with the participants’ needs, the right to self-determination and respect for human dignity.

**Approach**: The participants are offered the opportunity to be interviewed in a suitable environment at Akershus University Hospital, but they decide for themselves where the interviews will take place.

| **Needs and eventual risks** | **Interventions** |
| --- | --- |
| Discomfort during interview | Suggest breaks  Give the participant the opportunity to cancel or postpone the interview |
| Stronger reaction during the interview | Based on the principle of "I see you; I understand you; I accept you," use validation as a technique to convey empathy and unconditional acceptance.  Use normalization techniques (tell the participant that their reaction is normal). This is critical for the participant |
| Need for specialized help during the interview | Acute psychiatry department at Akershus University hospital (Ahus) assists with guidance and further planning  Project contact: Juha Tapio Silvola (section chief physician, Ahus)  Phone number: xxxxxxxx |
| Follow-up after the interview, short-term follow-up | Interviewers contact the participant to identify the need for help:  1. one day after the interview  2. one week after the interview  If there is a need for specialized help, the interviewer contacts the emergency psychiatric unit (Ahus) for guidance and advice on further plans.  Participants receive the research group's telephone number and contact number for the Acute psychiatry-department at Ahus.  Ana Carla Schippert: xxxxxxxx  Ann Kristin Bjørnnes: xxxxxxxx  Ellen Karine Grov: xxxxxxxx  Bente Nilsen: xxxxxxxx |
| Follow-up after the interview, long-term follow-up | The participants receive the research group's contact details. Participants can contact the members of the research group for one month after the interview.  Ana Carla Schippert: xxxxxxxx  Ann Kristin Bjørnnes: xxxxxxxx  Ellen Karine Grov: xxxxxxxx  Bente Nilsen: xxxxxxxx |
| Follow-up to relatives | The participants and the next of kin receive the research group's contact details and can contact the members of the research group for one month after the interview.  Ana Carla Schippert: xxxxxxxx  Ann Kristin Bjørnnes: xxxxxxxx  Ellen Karine Grov: xxxxxxxx  Bente Nilsen: xxxxxxxx  If there is a need for specialized help, the interviewer contacts the acute psychiatric unit (Ahus) for guidance and advice on further plans. |
