# Supplementary material for: Torture survivors’ experiences of receiving surgical treatment indicating re- traumatization
Source: PLoS One. 2023 Oct 17;18(10):e0287994. doi: 10.1371/journal.pone.0287994 (PMC10581467; doi:10.1371/journal.pone.0287994)
Supplement: S1 Table — (DOCX) [file pone.0287994.s006.docx]

**Supplemental table 1.** **Overview of the participants, the described torture methods, context and sequels, type of treatment, treatments context, and healthcare providers involved in the treatment.**

| Participants  (fictitious name | Torture methods | Context and torturer | Sequels | Treatment | Treatments context | Healthcare providers involved in treatment |
| --- | --- | --- | --- | --- | --- | --- |
| P1 | . Imprisoned for 35 months in late adolescence  . Interrogation  . Blindfolded  . Punched all over the body and head  . Electric shock  . Isolation  . 20 days in a damp and a small cell  . Waterboarding  . Listened to others being tortured  . Harsh beatings to the ears  . Death threats  . Suspension for hours | . Police station  . Prison  . Police officer  . Military | . Ear damage  . Rupture of the tympanic membrane  . Bleeding from ears  . Pain | . Heart (twice)  . Testicle | . Hospital  . Operating theater  **.** Intensive care unit | . Surgeon  . Anesthesiologist  . Nurses |
| P2 | . Imprisoned for 5 years when he was 23 years old  . Blindfolded  . Interrogation  . Electric shocks on the tongue, testicles, and anus  . Darkness (deprivation of light)  . Isolation  . No access to a toilet  . A friend was killed in prison.  . Brother also imprisoned  . Death attempts  . Suspension (hanging by the legs)  . Waterboarding  . Food deprivation  . Threats | . Tortured at the hospital when he was lying on bed  . Doctors involved in torture  . Prison  . Some of the torturers were friends of the family | . Urinating blood  . Bloody stool  . Pain  . Swollen head  . Back injury | . Subject to surgical treatment 12 times  . Heart surgery  . Knee (arthroscopy and open surgery) | . Hospitals  . Operating theater  . Postoperative unit | . Surgeons  . Anesthesiologists  . Nurses |
| P3 | . Deprivation of food and water  . He saw many people die  . Death threats  . Gun pointed at his head  . Blindfolded  . Beatings on shoulders and head  . Hands and legs tied | . Refugee camp  . Confined  . Tortured by militants | . Memory problems  . Pain all over his body  . Problems walking | . Eyes (twice)  . Shoulder (twice)  . Heart (twice) | . Hospital  . Operating theater  . Postsurgical department | . Surgeon  . Anesthesiologist  . Nurses |
| P4 | . Interrogation  . Beating and kicking (face, crotch, stomach, ribs, all over the body]  . Bent fingers backward  . Brought in for torture during meals  . Exposed to extreme cold  . Body search as well as all cavities of the body  . Death threats  . Witnessed others being tortured  . Naked  . Blindfolded  . Tied to a bed  . Surgical or painful treatment without local anesthetic  . In a cramped cell (positional torture)  . Unpredictability  . Children were killed  . Threats against the family  . Two brothers were also imprisoned | . Arrested by a schoolmate from childhood  . Prison  . Tortured by a distant relative  . Dark cell  . A doctor collaborated with torturers | . Pain in the bones of the hand  . Pain all over his body  . Hemorrhoids | Hemorrhoidectomy (twice) | . Hospital  . Operating theater  . Postsurgical department | . Surgeon  . Anesthesiologist  . Nurses |
| P5 | . Imprisoned for 11 years  . Interrogation  . Blindfolded  . Death threats  . Beating under the foot with an electric cable  . Tossed back and forth  . Kick in the back, head, face, and whole body  . Black bag over head  . Tied to a bed  . Mock execution  . Stretching of his body while he was tied to a bed  . Feet bent backward  . Face burnt with a lighter  . Water torture  . Twisting of the testicles  . “One guard sat on my back.”  . Forced positions  . Harsh beatings to the ears (telefono)  . Strangulation  . Suspension for 17 hours  . Threatened with a gun pointed at his head  . Fake execution  . Blows to teeth  . In a cramped cell for 5 months  . Completely dark cell (deprivation of light)  . Extreme heat  . Isolation  . Deprivation of water and food  . Received the death penalty  . Other prisoners were executed  . Waited for execution every night for 6 months | . A doctor collaborated with the military forces  . A school principal collaborated with the torturers | . Blood in the urine  . Broken back  . Pain all over his body  . Broken teeth  . Burns on the knees  . Dehydration | . Extirpation of skin cancer on the head  . Assessment of back pain (for eventual surgery) | . Hospital  Emergency | . Surgeon  . Nurses  . Healthcare secretary |
| P6 | . Imprisonment  . Lack of water  . Combat situation  . Body beaten  . Sexual humiliation  . Extortion  . Forced to hide  . Kidnapped  . Isolation  . Family member killed  . Witness beatings to head and body  Witness murder | . Prison  . Combat | . Chronic pain in the whole body | . Nose  . Pain (suspicion of hernia inguinalis) | . Surgical outpatient clinic  . Surgical bed post for 3 days | . Nurses  . Surgeons  . Physicians |
| P7 | . Deprivation of water and food  . Sexual torture  . Blows and kicks  . Beating to the body  . Stabbed  . Kidnapped  . Family member killed  . Family member tortured  . Electric shocks | . Prison  . Combat | . Pain  . Back problems  . Damaged teeth | . Examination of hemorrhoids and rectal bleeding without any kind of local anesthesia (once)  . Examination of hemorrhoids and rectal bleeding under local anesthesia (twice).  . Assessment of back pain (for eventual surgery) | . Surgical outpatient clinic  . Operating theater | . Nurses  . Surgeons  . Physicians |
| P8 | . Lack of food and water  . Beatings to the head and back with a cable  . Falanga  . Suspension  . Witness others’ torture | . Prison | . Pain  . Back problems | . Back surgery (six times) | . Operating theater | . Nurses  . Surgeons |
